# De novo transcriptome analysis and comparative expression profiling of genes associated with the taste-modifying protein neoculin in *Curculigo latifolia* and *Curculigo capitulata* fruits

**DOI:** 10.1186/s12864-021-07674-3

**Published:** 2021-05-13

**Authors:** Satoshi Okubo, Kaede Terauchi, Shinji Okada, Yoshikazu Saito, Takao Yamaura, Takumi Misaka, Ken-ichiro Nakajima, Keiko Abe, Tomiko Asakura

**Affiliations:** 1 The Yamashina Botanical Research Institute, Nippon Shinyaku Co., Ltd., Oyake Sakanotsuji-cho 39, Yamashina-ku, Kyoto, 607-8182 Japan; 2 Graduate School of Agricultural and Life Sciences, The University of Tokyo, 1-1-1, Yayoi, Bunkyo-ku, Tokyo, 113-8657 Japan; 3 Present address: Division of Endocrinology and Metabolism, National Institute for Physiological Sciences, 38 Nishigonaka, Myodaiji, Okazaki, Aichi 444-8585 Japan; 4 Kanagawa Institute of Industrial Science and Technology (KISTEC), 3-25-13 Tonomachi, Kawasaki-ku, Kawasaki, Kanagawa 210-0821 Japan

**Keywords:** NGS, RNA-seq, Neoculin, NBS, NAS, *Curculigo capitulata*, *Curculigo latifolia*, Expression profile, Gene duplication

## Abstract

**Background:**

*Curculigo latifolia* is a perennial plant endogenous to Southeast Asia whose fruits contain the taste-modifying protein neoculin, which binds to sweet receptors and makes sour fruits taste sweet. Although similar to snowdrop (*Galanthus nivalis*) agglutinin (GNA), which contains mannose-binding sites in its sequence and 3D structure, neoculin lacks such sites and has no lectin activity. Whether the fruits of *C. latifolia* and other *Curculigo* plants contain neoculin and/or GNA family members was unclear.

**Results:**

Through de novo RNA-seq assembly of the fruits of *C. latifolia* and the related *C. capitulata* and detailed analysis of the expression patterns of *neoculin* and *neoculin-like* genes in both species, we assembled 85,697 transcripts from *C. latifolia* and 76,775 from *C. capitulata* using Trinity and annotated them using public databases. We identified 70,371 unigenes in *C. latifolia* and 63,704 in *C. capitulata*. In total, 38.6% of unigenes from *C. latifolia* and 42.6% from *C. capitulata* shared high similarity between the two species. We identified ten *neoculin*-related transcripts in *C. latifolia* and 15 in *C. capitulata*, encoding both the basic and acidic subunits of neoculin in both plants. We aligned these 25 transcripts and generated a phylogenetic tree. Many orthologs in the two species shared high similarity, despite the low number of common genes, suggesting that these genes likely existed before the two species diverged. The relative expression levels of these genes differed considerably between the two species: the transcripts per million (TPM) values of *neoculin* genes were 60 times higher in *C. latifolia* than in *C. capitulata*, whereas those of GNA family members were 15,000 times lower in *C. latifolia* than in *C. capitulata*.

**Conclusions:**

The genetic diversity of *neoculin*-related genes strongly suggests that *neoculin* genes underwent duplication during evolution. The marked differences in their expression profiles between *C. latifolia* and *C. capitulata* may be due to mutations in regions involved in transcriptional regulation. Comprehensive analysis of the genes expressed in the fruits of these two *Curculigo* species helped elucidate the origin of neoculin at the molecular level.

**Supplementary Information:**

The online version contains supplementary material available at 10.1186/s12864-021-07674-3.

## Background

*Curculigo latifolia* (Hypoxidaceae family, formerly classified in the Liliaceae family) is a perennial plant found in Southeast Asia, especially the Malay peninsula [1, 2]. According to the Royal Botanic Gardens, Kew, there are 27 species of *Curculigo* [3]. The genetic diversity and morphology of *Curculigo* have long been of interest [4–7]. *C. latifolia* and *C. capitulata* were previously reclassified as members of the *Molineria* genus, but recent discussions have suggested that they should be returned to the *Curculigo* genus. Here, we use the traditional name, *Curculigo*.

**Fig. 1:**
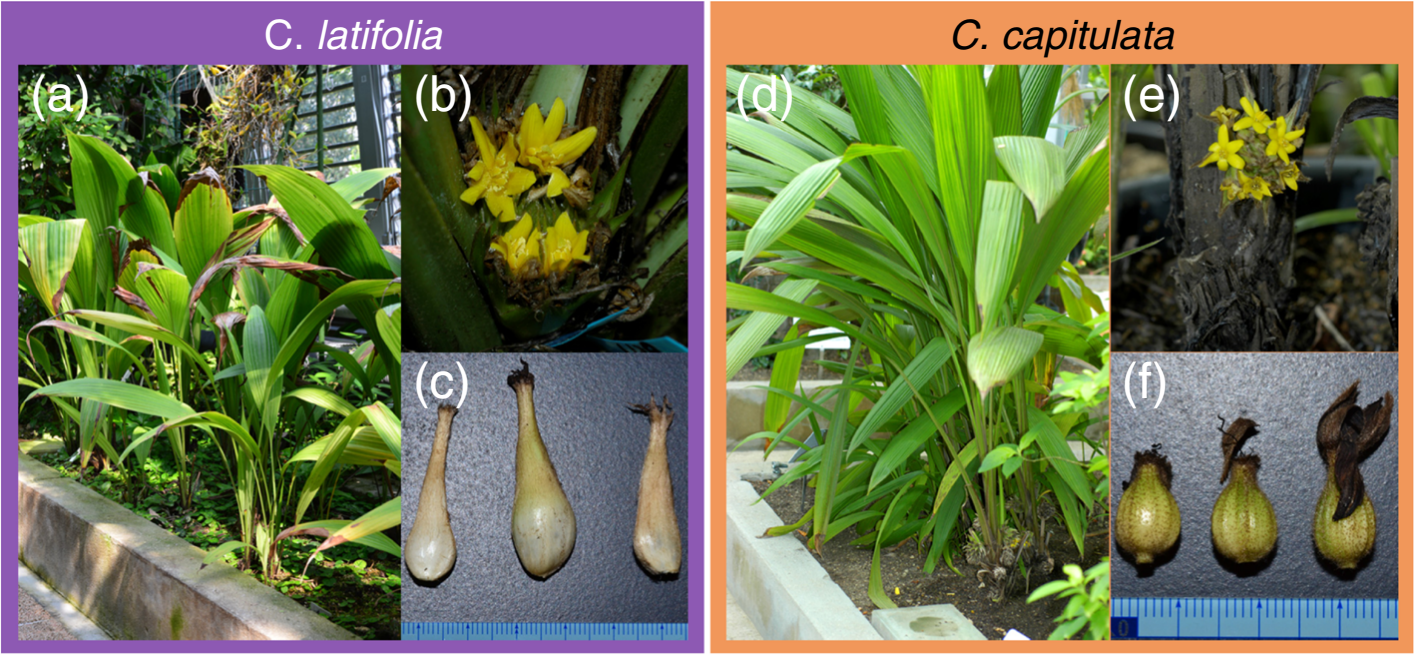
Photographs of *Curculigo latifolia* and *Curculigo capitulata*. *Curculigo latifolia* (**a**–**c**) and *C. capitulata* (**d**–**f**) in the greenhouse at the Yamashina Botanical Research Institute. **b** and **e** Inflorescences; **c** and **f** fruits. All photographs are our own taken by Satoshi Okubo

*C. latifolia* and *C. capitulata* have a similar vegetative appearance (Fig. 1), but differ in their flower and fruit morphology. In addition, *C. capitulata* is more widely distributed than *C. latifolia*. Both species are diploids (2*n* = 18; *x* = 9) [8]. *C. latifolia* is self-incompatible [9], but *C. capitulata* plants from various botanical gardens in Japan have not been successfully crossed. So, it is unknown whether *C. capitulata* is self-compatible or self-incompatible. The flowers, roots, stems, and leaves of *Curculigo* plants have traditionally been used as medicines [10–15]. Notably, *C. latifolia* fruits, but not those of *C. capitulata*, produce a taste-modifying protein, neoculin, that makes sour-tasting foods or water taste sweet [1, 16–18].

Neoculin itself has a sweet taste and is 550 times sweeter than sucrose on the percentage sucrose equivalent scale [19, 20]. Furthermore, neoculin has a taste-modifying activity that converts sourness to sweetness: for example, the sour taste of lemons is changed to a sweet orange taste. Moreover, the presence of neoculin induces sweetness in drinking water, and some organic acids taste sweet when consumed after neoculin [21]. Neoculin is perceived by the human sweet taste receptor T1R2-T1R3, a member of the G-protein-coupled receptor family [22]. Neoculin consists of two subunits that form a heterodimer: the neoculin basic subunit (NBS), also called curculin [16], and the neoculin acidic subunit (NAS) [18, 23]. NBS is a 11-kDa peptide consisting of 114 amino acid residues [16, 24], while NAS has a molecular mass of 13 kDa and 113 residues. The two subunits share 77% identity at the protein level [18]. Several essential amino acids that are responsible for the taste-modifying properties of neoculin have been identified: His-11 in NBS is responsible for the pH-dependent taste-modifying activity of neoculin [25], and Arg-48, Tyr-65, Val-72, and Phe-94 function in the binding and activation of human sweet taste receptors [26]. Changes in the tertiary structure of the subunits at these residues are thought to contribute to the taste-modifying properties of neoculin [27, 28].

Lectins are proteins that recognize and bind to specific carbohydrate structures [29, 30]. Plant lectins are classified into 12 families. Neoculin NBS and NAS are similar in protein sequence and 3-dimensional (3D) structure to the GNA (*Galanthus nivalis* agglutinin) family of lectins, which are present in bulbs such as snowdrop (*Galanthus nivalis*) and daffodil (*Narcissus pseudonarcissus*) and are thought to function as defense or storage proteins [31–33]. However, NBS and NAS lack a mannose-binding site (MBS) and do not have lectin activity [34–36]. Furthermore, whereas GNA family members in plants such as snowdrop contain one disulfide bond, which functions in intra-subunit bonding, neoculin forms both two intra-subunit bonds and two inter-subunit bonds between NBS and NAS [32].

The fruit of *C. latifolia* contains 1.3 mg neoculin per fruit [37] or 1.3 mg per one gram of fresh pulp [38]. This is thought to be considerably higher than the levels of total proteins in typical edible fruits [39]. Although the taste-modifying activity of neoculin is well-known, its biological role in *C. latifolia* is unknown. In addition, as neoculin is not a lectin, it was not clear which lectins are expressed in *C. latifolia* fruits, especially lectins of the GNA family. Finally, whether other *Curculigo* species also accumulate neoculin or neoculin-like proteins is unknown.

Here, we compared the gene expression profiles in the fruits of *C. latifolia* and *C. capitulata* by transcriptome deep sequencing (RNA-seq). The aim of this study was to comprehensively analyze the two species from the viewpoint of amino acid sequences and gene expression levels to shed light on the origins of neoculin.

## Results

### De novo RNA-seq assembly from *C. latifolia* and *C. capitulata* fruits

We sequenced cDNA libraries from *C. latifolia* and *C. capitulata* using the Illumina HiSeq 2500 platform. To analyze the data, we filtered out raw reads with average quality values < 20, reads with < 50 nucleotides, and reads with ambiguous ‘N’ bases. After trimming reads for adapter sequences and filtering, we obtained 44,396,896 reads from *C. latifolia* and 43,863,400 from *C. capitulata*. We then assembled high-quality reads from *C. latifolia* and *C. capitulata* into 85,697 and 76,775 contigs with a mean length of 775 bp and 744 bp, respectively, using Trinity 2.11. The distribution of transcript lengths and transcripts per million (TPM) values are shown in Additional files 1 and 2. The N50 values for *C. latifolia* and *C. capitulata* transcripts were 1324 and 1205, respectively (Table 1). Unigene clustering using CD-Hit revealed 70,371 unigenes in *C. latifolia* and 63,704 in *C. capitulata* (Table 1)*.*

**Table 1:** Overview of de novo RNA-seq assembly from *C. latifolia* and *C. capitulata* fruits

	***C. latifolia***	***C. capitulata***
**High-quality reads**	44,396,896	43,863,400
**Total Trinity genes**	69,446	63,951
**Total Trinity unigenes**	70,371	63,704
**Total Trinity transcripts**	85,697	76,775
**GC (%)**	44.0	45.6
**N10 (nts)**	3214	2676
**N20 (nts)**	2460	2103
**N50 (nts)**	1324	1205
**Total assembled bases**	66,426,868	57,098,016

### The gene repertoires of the two *Curculigo* species fitting the monocots

Low annotation rate of the transcripts: To gather functional information about the transcripts identified from de novo assembly, we aligned all transcripts against nucleotide sequences from various protein databases, including the nonredundant protein (NR) database at the National Center for Biotechnology Information (NCBI), RefSeq, UniProt/Swiss-Prot, Clusters of Orthologous Groups of proteins (COG), the rice (*Oryza sativa*) genome (Os-Nipponbare-Reference-IRGSP-1.0, Assembly: GCF_001433935.1), and the Arabidopsis (*Arabidopsis thaliana*) genome (Assembly: GCF_000001735.4) and selected the top hits from these queries. We obtained annotations for 38,433 out of 85,697 transcripts (44.8%) in *C. latifolia* and 40,554 out of 76,775 transcripts (52.8%) in *C. capitulata* with a threshold of 1e^− 10^ by performing a Basic Local Alignment Search Tool search with our in silico-translated transcripts against protein databases (BLASTx) using the NR, RefSeq, UniProt, and COG databases and the proteomes of rice and Arabidopsis. All annotations are listed in Additional file 3. The number of annotated transcripts for each database is listed in Table 2. The low annotation rate suggests that the two *Curculigo* species are significantly different from classical model plant systems that drive much of the information stored in public databases.

**Table 2:** Number of functional annotations of transcripts from *C. latifolia* and *C. capitulata* fruits

Annotated database	***C. latifolia***	***C. capitulata***
**COG**^a^	11,875	12,448
**RefSeq**	37,922	39,369
**Uniprot**	36,783	38,901
**NR**^b^	37,118	39,340
**Rice**^c^	34,761	36,204
**Arabidopsis**^d^	33,332	34,684
**All six databases**	38,433	40,554

^a^*COG* Clusters Groups of proteins

^b^*NR* nonredundant protein databases of the National Center for Biotechnology Information

^c^Assembly: GCF_001433935.1

^d^Assembly: GCF_000001735.4

**Fig. 2:**
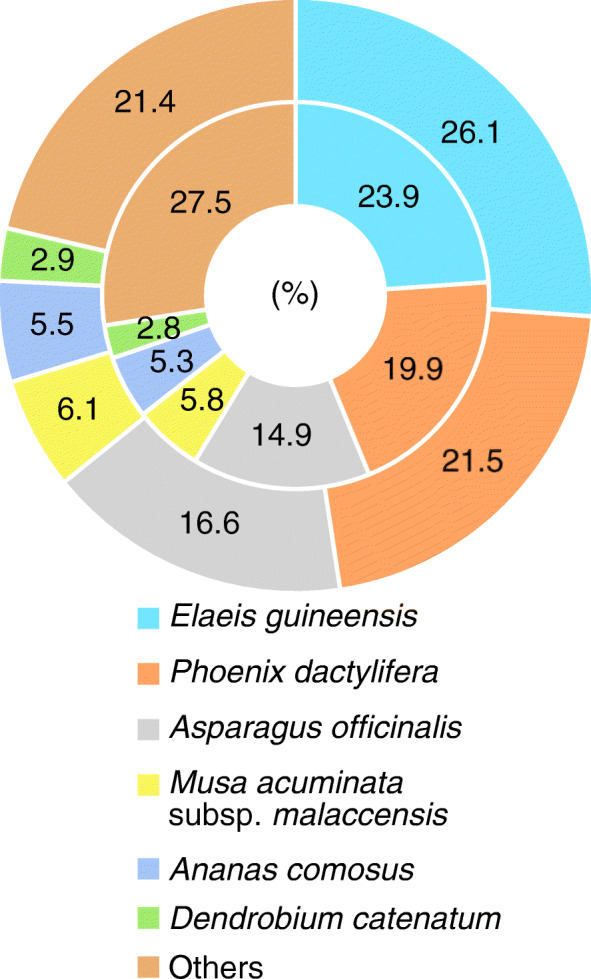
The de novo assembled *C. latifolia* and *C. capitulata* transcriptomes reveal high similarity to known monocot genes. The percentage of genes with matches in *C. latifolia* (outer circle) and *C. capitulata* (inner circle) was obtained from the results of BLAST search against the NR database. The top six most highly homologous species were monocot, like *Curculigo*

Conservation across monocots: After BLASTx searches with the *C. latifolia* and *C. capitulata* transcripts against the NR database, we determined the extent of gene conservation across plant species by running Blast2GO [40]. We estimated the similarity of the two *Curculigo* species to various plant species by counting the number of hits from each species obtained by BLAST searches (Fig. 2). The top six species displaying the highest homology with *C. latifolia* and *C. capitulata* transcripts were monocots, like *Curculigo*, supporting the view that the assembled *Curculigo* genes are highly similar to known genes from other monocots. The top six species sharing the highest similarity with *C. latifolia* and *C. capitulata* were identical in terms of both species and rank order.

**Fig. 3:**
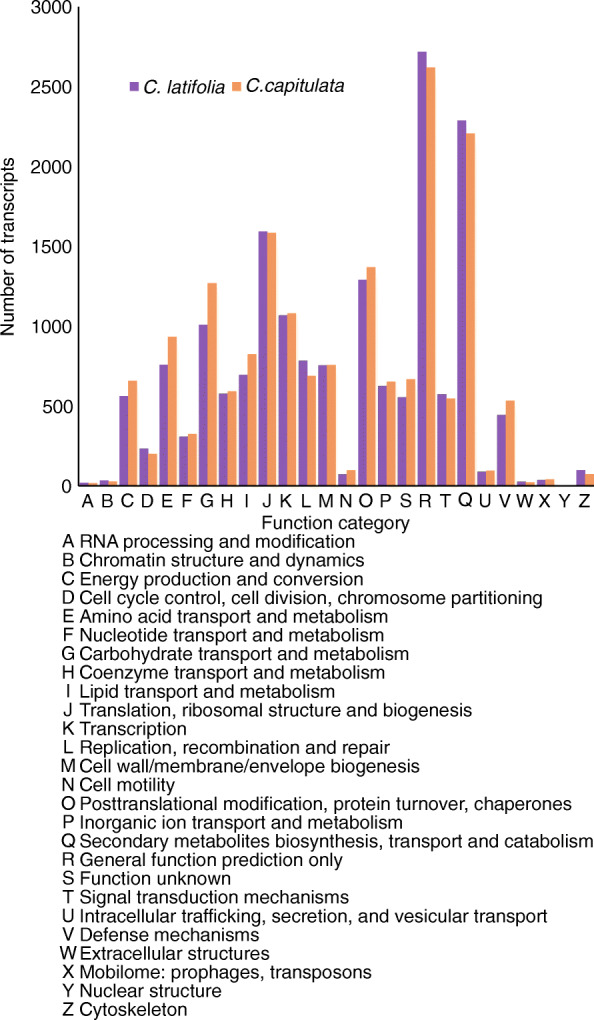
*C. latifolia* and *C. capitulata* have functionally similar genes. Functional classification of transcripts was performed using the COG database. In total, 11,875 (*C. latifolia*) and 12,448 (*C. capitulata*) transcripts were grouped into 26 COG categories (A to Z). No significant differences were observed between the two species

Expression of functionally similar genes between the two species: Using the COG database, we classified 11,875 transcripts from *C. latifolia* and 12,448 from *C. capitulata* into functional categories (Fig. 3). We observed no significant differences between the two species, which supports the notion that these two species have functionally similar genes.

We also analyzed the functions of the assembled transcripts via Gene Ontology (GO) analysis using the rice genome annotation (Additional file 4). Again, no significant differences were observed between the two species. The results also suggested that the repertoires of genes from the two species are similar to those of better-known species.

### The genes with high similarity between *C. latifolia* and *C. capitulata* fruits are less than half of the genes

**Fig. 4:**
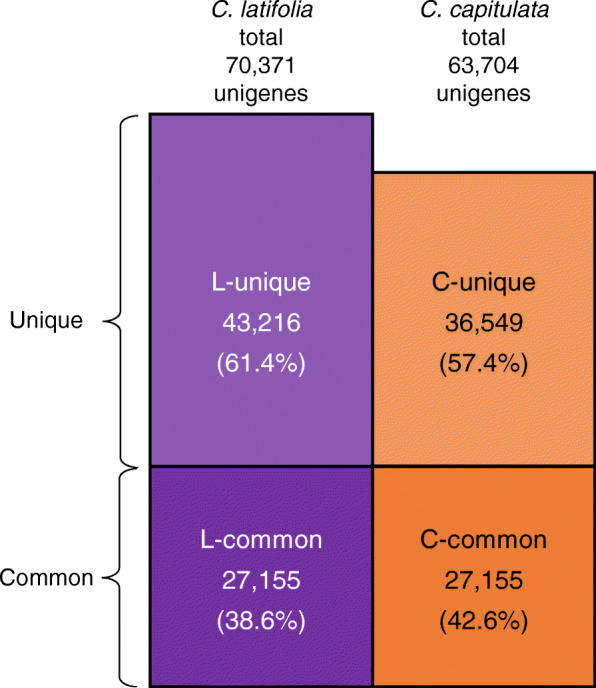
The majority of unigenes from *C. latifolia* and *C. capitulata* correspond to unique genes with low similarity. Number of unigenes based on sequence similarity between *C. latifolia* and *C. capitulata* fruits. The number of highly similar unigenes that are common (L-common: common genes of *C. latifolia*; C-common: common genes of *C. capitulata*) and unigenes with low similarity, which are thus unique genes (L-unique: unique genes of *C. latifolia*; C-unique: unique genes of *C. capitulata*)

Using the unigene sequences, we analyzed the similarity of between *C. latifolia* and *C. capitulata* genes. We performed BLAST searches using each transcript from one species as the query sequence against all transcripts from the other species with a threshold *E*-value of 1e^− 5^ or less and selected the reciprocal best hits. We defined unigenes with high similarity between the two species as common genes and unigenes with low similarity between the species, or present in only one species, as unique genes. In total, we deemed 38.6% (27,155 out of 70,371) of genes in *C. latifolia* and 42.6% (27,155 out of 63,704) of genes in *C. capitulata* to be common genes (Fig. 4). The relatively small number of common genes suggests that a long time has passed since the divergence of these species, which is consistent with results of lineage analysis based on plastid DNA from Hypoxidaceae family members. Indeed, although the *Curculigo* genus constitutes a single clade, *C. latifolia* and *C. capitulata* are not the most closely related species within this clade [5]*.*

Next, we investigated the proportion of annotated genes in these species using the COG, RefSeq, UniProt, and NR databases and the genomes of rice and Arabidopsis (shown in Table 2). Among the common genes, 17,337 and 17,199 genes were annotated (63.8 and 63.3% of common genes) in *C. latifolia* and *C. capitulata*, respectively. By contrast, there were 11,718 annotated unique genes (27.1% of unique genes) among genes found only in *C. latifolia* and 14,848 (40.6% of unique genes) among those found only in *C. capitulata*. Thus, the annotation rate was higher for common genes than for unique genes, despite the smaller number of common genes. One possible explanation for this observation is that many of the genes common to both species may also be common genes in other model plant species that are highly represented in the databases employed.

We then compared the expression profiles of 27,155 common genes between *C. latifolia* and *C. capitulata*. Although the sequences of the corresponding genes in *C. latifolia* and *C. capitulata* were similar, their expression profiles were not necessarily equivalent. Nonetheless, only 111 out of the 27,155 common genes had TPM ratios ≥50 (Table 3). Of these 111 genes, five were *neoculin*-related genes, indicating that the expression profiles of at least some *neoculin*-related genes differ significantly between the two species.

**Table 3:** Comparison of the expression profiles of *C. latifolia* and *C. capitulata*

***C. latifolia***			***C. capitulata***				
Transcript ID	RefSeq	TPM	Transcript ID	RefSeq	TPM	Pident^a^	***E***-value^a^
L_19492_c6_g1_i1	trans-resveratrol di-O-methyltransferase	36,282	C_19332_c0_g2_i1	trans-resveratrol di-O-methyltransferase	277	99.18	0
L_20774_c6_g2_i5	trans-resveratrol di-O-methyltransferase	31,648	C_20405_c1_g1_i2	trans-resveratrol di-O-methyltransferase	573	99.02	0
*L_22219_c0_g1_i1	mannose-specific lectin-like	7634	*C_16562_c0_g1_i1	mannose-specific lectin-like	80	97.75	0
L_22040_c0_g1_i1	chalcone synthase-like	6483	C_22230_c0_g1_i1	chalcone synthase-like	69	100	0
L_39489_c0_g1_i1	cinnamoyl-CoA reductase 1-like	4584	C_43958_c0_g1_i1	cinnamoyl-CoA reductase 1-like	37	100	0
L_17418_c0_g1_i1	benzyl alcohol O-benzoyltransferase	2848	C_20771_c2_g1_i3	benzyl alcohol O-benzoyltransferase	18	96.27	0
L_18625_c0_g1_i1	glutelin type-A 1-like	2641	C_18515_c0_g1_i1	glutelin type-A 1-like	35	100	0
L_20161_c0_g1_i1	probable polyamine oxidase 5	2333	C_20921_c0_g1_i1	probable polyamine oxidase 5	38	99.17	0
L_20171_c0_g1_i1	pyruvate decarboxylase 1 isoform X1	2140	C_19622_c0_g1_i1	pyruvate decarboxylase 1 isoform X1	30	99.74	0
L_19390_c0_g1_i1	benzyl alcohol O-benzoyltransferase-like	1721	C_20336_c0_g1_i1	benzyl alcohol O-benzoyltransferase-like	25	99.01	0
L_17288_c0_g1_i1	5-methyltetrahydropteroyl-triglutamate--homocysteine methyltransferase 1	1527	C_20491_c0_g1_i4	5-methyltetrahydropteroyl-triglutamate--homocysteine methyltransferase 2-like	19	98.22	0
L_22101_c0_g1_i1	cytochrome P450 71A1-like	1130	C_20591_c0_g1_i1	cytochrome P450 71A1-like	14	100	0
L_9054_c0_g2_i1	uncharacterized protein LOC105052971	891	C_20462_c0_g1_i1	uncharacterized protein LOC105052971	16	99.15	0
L_19899_c1_g1_i5	elongation factor 1-alpha-like	720	C_16211_c0_g1_i1	hypothetical protein CARUB_v100096370mg, partial	11	99.75	0
L_39417_c0_g1_i1	palmitoyl-acyl carrier protein thioesterase, chloroplastic-like	659	C_1125_c0_g1_i1	palmitoyl-acyl carrier protein thioesterase, chloroplastic-like	0.89	99.88	0
L_8999_c0_g1_i1	probable protein Pop3	657	C_3239_c0_g1_i1	probable protein Pop3	10	99.79	0
*L_16562_c0_g1_i1	mannose-specific lectin-like	652	*C_16324_c0_g1_i1	mannose-specific lectin-like	8	98.8	0
L_20784_c0_g1_i1	mannan endo-1,4-beta-mannosidase 5-like	477	C_20300_c0_g1_i1	mannan endo-1,4-beta-mannosidase 5-like	8	99.81	0
L_17063_c0_g1_i1	uncharacterized protein LOC103705182	457	C_15604_c0_g1_i1		7	99.51	0
L_9763_c0_g1_i1	4-hydroxyphenyl-pyruvate dioxygenase	441	C_17419_c0_g1_i2	4-hydroxyphenyl-pyruvate dioxygenase	6	97.85	0
L_15645_c0_g1_i1	hypothetical protein PHAVU_005G042200g	378	C_19503_c0_g1_i2	uncharacterized protein LOC103713005	4	98.6	0
L_39500_c0_g1_i1	uncharacterized protein C24B11.05-like isoform X2	323	C_15665_c0_g1_i2	uncharacterized protein C24B11.05-like isoform X2	6	96.74	0
L_16206_c0_g1_i1	cytochrome P450 71A1-like	295	C_18399_c0_g1_i1	cytochrome P450 71A1-like	5	99.88	0
L_9770_c0_g1_i1	Os09g0480700, partial	278	C_11365_c0_g1_i1	Os09g0480700, partial	3	99.52	0
L_20943_c2_g1_i1	LOW QUALITY PROTEIN: ATP-citrate synthase beta chain protein 1-like	276	C_20189_c1_g1_i6	LOW QUALITY PROTEIN: ATP-citrate synthase beta chain protein 1-like	5	99.74	0
L_5031_c0_g1_i1		265	C_26197_c0_g1_i1		3	99.53	2E-108
L_19581_c0_g1_i1	peroxidase 43	244	C_20763_c0_g1_i7	peroxidase 43	3	99.32	0
L_22200_c0_g1_i1		237	C_21279_c0_g3_i1		0	92.42	0
L_16082_c0_g1_i1	uncharacterized protein LOC105035694	230	C_20815_c0_g1_i2	uncharacterized protein LOC105035694	4	97.73	0
L_1821_c0_g1_i1	protein EARLY RESPONSIVE TO DEHYDRATION 15-like	213	C_5863_c0_g3_i1	protein EARLY RESPONSIVE TO DEHYDRATION 15-like	1	94.17	0
L_21840_c4_g7_i1		197	C_46444_c0_g1_i1		2	100	0
L_11489_c0_g1_i1		189	C_51079_c0_g1_i1		3	95.13	4E-114
L_21813_c0_g1_i1	protein kinase APK1B, chloroplastic-like	184	C_20869_c0_g1_i9	protein kinase APK1B, chloroplastic-like	0.97	95.04	0
L_16611_c0_g1_i1		163	C_8161_c0_g1_i1		2	100	0
L_12355_c0_g1_i1	myb-related protein 306-like	160	C_7266_c0_g1_i1	myb-related protein 306-like	3	99.89	0
L_18378_c0_g1_i1	probable L-ascorbate peroxidase 4	158	C_17994_c1_2_i1	probable L-ascorbate peroxidase 4	2	96.39	0
L_21677_c0_g1_i1	S-adenosylmethionine decarboxylase proenzyme-like	149	C_15562_c0_g2_i1	S-adenosylmethionine decarboxylase proenzyme-like	0.92	96.67	0
L_14830_c0_g1_i1	NAC transcription factor 29-like	135	C_20428_c0_g1_i1	NAC transcription factor 29-like	0	97.61	0
L_14165_c0_g2_i1	probable peroxygenase 4	131	C_17339_c0_g1_i2	probable peroxygenase 4	2	95.32	0
L_21840_c4_g4_i2		130	C_11729_c0_g1_i1		2	100	0
L_39737_c0_g1_i1	Glutathione peroxidase 2	127	C_8347_c0_g1_i1	Glutathione peroxidase 2	2	96.26	0
L_4928_c0_g1_i1		124	C_44794_c0_g1_i1		1	100	3E-101
L_20250_c0_g1_i1	protein NRT1/ PTR FAMILY 5.6-like	114	C_29979_c0_g1_i1	protein NRT1/ PTR FAMILY 5.6-like	2	97.44	0
L_15628_c0_g1_i1	formin-A-like	103	C_20575_c0_g1_i5	formin-A-like	0	90.44	0
L_21235_c2_g9_i1		101	C_9877_c0_g1_i1		1	92.77	3E-98
*L_19752_c0_g1_i1	mannose-specific lectin 3-like	33	*C_18595_c0_g1_i1	mannose-specific lectin 3-like	2301	97.6	0
L_16463_c0_g2_i2	LOW QUALITY PROTEIN: S-norcoclaurine synthase-like	16	C_6989_c0_g1_i1	LOW QUALITY PROTEIN: S-norcoclaurine synthase-like	8393	91.39	0
L_32395_c0_g1_i1		14	C_4973_c0_g1_i1		8765	86.17	4E-92
L_19456_c0_g1_i1	polyphenol oxidase, chloroplastic-like	13	C_20237_c3_g1_i1	polyphenol oxidase, chloroplastic-like	1496	83.82	0
L_14333_c0_g1_i1		12	C_13197_c0_g1_i1		42,047	94.54	7E-75
L_55067_c0_g1_i1	defensin Ec-AMP-D1 {ECO:0000303| PubMed:18625284}-like	9	C_39416_c0_g1_i1	defensin Ec-AMP-D1 {ECO:0000303| PubMed:18625284}-like	2475	95.1	0
L_5253_c0_g1_i1	Disease resistance-responsive (dirigent-like protein) family protein, putative	9	C_16870_c0_g2_i1	Disease resistance-responsive (dirigent-like protein) family protein, putative	547	94.54	0
L_1586_c0_g1_i1	glycine-rich protein-like isoform X1	8	C_39384_c0_g1_i1		2895	94.72	0
L_23556_c0_g1_i1	basic blue protein-like	5	C_14117_c0_g1_i1	basic blue protein-like	606	94.75	0
L_13618_c0_g1_i1	non-specific lipid-transfer protein 1-like	5	C_13976_c0_g1_i1	lipid transfer protein precursor	655	96.6	0
L_465_c0_g2_i1	microsomal glutathione S-transferase 3-like	5	C_4959_c0_g1_i1	microsomal glutathione S-transferase 3-like	246	93.89	0
L_21384_c3_g4_i1		5	C_17484_c0_g1_i1		424	86.76	1E-56
L_9003_c0_g1_i1	dirigent protein 22-like isoform X1	5	C_19511_c0_g1_i1	dirigent protein 22-like	834	96.09	0
L_4015_c0_g1_i1	CASP-like protein 2A1	4	C_4840_c0_g1_i1	CASP-like protein 2A1	241	98.25	0
L_16618_c0_g1_i1	hypothetical protein SORBIDRAFT_05g026700	3	C_4999_c0_g1_i1	Bowman-Birk type trypsin inhibitor-like isoform X2	5459	86.06	1E-135
L_4834_c0_g2_i1	xylem serine proteinase 1-like	3	C_9966_c0_g1_i1	subtilisin-like protease	232	96.91	0
L_6907_c0_g1_i1	serine/threonine-protein kinase CDL1-like	3	C_11871_c1_g1_i1	serine/threonine-protein kinase CDL1-like	183	96.44	0
L_17444_c0_g1_i1	cytochrome P450 CYP82D47-like	3	C_20684_c0_g1_i1	cytochrome P450 CYP82D47-like	182	94.92	0
L_40485_c0_g3_i1	non-specific lipid-transfer protein 1-like	3	C_39065_c0_g1_i1	non-specific lipid-transfer protein 1-like	1455	90.92	0
L_18380_c0_g1_i2	conserved hypothetical protein	3	C_39186_c0_g1_i1	conserved hypothetical protein	654	93.2	4E-127
L_31252_c0_g1_i1		3	C_12650_c0_g1_i1	non-specific lipid-transfer protein-like	283	94.9	4E-65
L_42464_c0_g2_i1	alpha carbonic anhydrase 8-like, partial	3	C_40148_c0_g1_i1	alpha carbonic anhydrase 7-like	235	93.1	0
L_13852_c0_g1_i1	endoglucanase 6	3	C_18579_c0_g1_i1	endoglucanase 19-like	707	97.65	0
L_39898_c0_g1_i1	oxygen-evolving enhancer protein 3–1, chloroplastic-like	2	C_12932_c0_g1_i1	oxygen-evolving enhancer protein 3–1, chloroplastic-like	160	96.93	0
L_6056_c0_g1_i1	Calvin cycle protein CP12–1, chloroplastic-like	2	C_12691_c0_g1_i1	calvin cycle protein CP12–1, chloroplastic	215	92.42	3E-128
L_6093_c0_g1_i1		2	C_41578_c0_g1_i1		170	93.87	2E-135
L_17773_c0_g1_i1	uncharacterized protein LOC105056845	2	C_12165_c0_g1_i1	uncharacterized protein LOC105056845	249	92.36	0
L_24151_c0_g1_i1	ribonuclease 3-like	2	C_39292_c0_g1_i1	ribonuclease 3-like	389	98.07	0
L_8678_c0_g1_i1	uncharacterized protein LOC105056672	2	C_4730_c0_g1_i1	uncharacterized protein LOC105056672	116	98.71	0
L_19431_c2_g4_i1	polyubiquitin 4-like, partial	2	C_20039_c0_g8_i1	hypothetical protein PHAVU_003G1236000g, partial	2116	94.38	6E-106
L_250_c1_g1_i1	probable glutathione S-transferase parA	2	C_16559_c0_g1_i1	probable glutathione S-transferase parA	419	98.26	0
L_10676_c0_g1_i1	probable linoleate 9S-lipoxygenase 5	2	C_17658_c0_g1_i1	probable linoleate 9S-lipoxygenase 5	1644	98.82	0
L_8975_c0_g1_i1	chitinase-like protein 1	2	C_16475_c1_g1_i1	chitinase-like protein 1	183	97.92	0
L_44393_c0_g1_i1	hypothetical protein POPTR_0004s03650g	2	C_18495_c2_g1_i1	conserved hypothetical protein	2751	92.78	3E-66
L_759_c0_g1_i1	CAS1 domain-containing protein 1-like	2	C_21365_c0_g1_i1	CAS1 domain-containing protein 1-like isoform X2	183	99.26	0
L_30327_c0_g1_i1	conserved hypothetical protein	2	C_23037_c0_g1_i1	conserved hypothetical protein	134	96.47	2E-116
L_5572_c0_g1_i1	short-chain type dehydrogenase/reductase-like	2	C_14194_c0_g1_i1	short-chain type dehydrogenase/reductase-like	205	96.22	0
L_45139_c0_g1_i1	putative germin-like protein 2–1	2	C_21890_c0_g1_i1	putative germin-like protein 2–1	111	96.2	1E-146
L_22251_c0_g1_i1	xyloglucan endotransglucosylase/ hydrolase protein 9-like	2	C_40495_c0_g2_i1	LOW QUALITY PROTEIN: xyloglucan endotransglucosylase/hydrolase protein 9-like	131	98.37	0
L_56341_c0_g1_i1	peroxidase 4-like	1	C_10149_c0_g1_i1	peptide-N4-(N-acetyl-beta-glucosaminyl) asparagine amidase A-like	1301	93.81	2E-93
L_56680_c0_g1_i1	peptide-N4-(N-acetyl-beta-glucosaminyl) asparagine amidase A-like	1	C_19920_c0_g1_i1	peroxidase 4-like	583	98.68	7E-113
*L_307_c0_g2_i1	mannose-specific lectin-like	1	*C_9931_c0_g1_i1	mannose-specific lectin-like	14,867	99.35	0
L_48085_c0_g1_i1	probable indole-3-acetic acid-amido synthetase GH3.1	1	C_19080_c1_g1_i1	probable indole-3-acetic acid-amido synthetase GH3.1	107	84.39	2E-70
L_46946_c0_g1_i1	chlorophyll a-b binding protein 7, chloroplastic-like	1	C_41884_c0_g1_i1	chlorophyll a-b binding protein, chloroplastic	141	98.41	0
L_4845_c0_g1_i1	chlorophyll a-b binding protein CP26, chloroplastic-like	1	C_10575_c0_g1_i1	chlorophyll a-b binding protein CP26, chloroplastic-like	353	98.25	0
L_23363_c0_g1_i1	uncharacterized protein LOC105056050	1	C_21609_c0_g1_i1	uncharacterized protein LOC105056050	1612	98.84	0
*L_30823_c0_g1_i1	mannose-specific lectin-like	1	*C_17363_c2_g1_i3	mannose-specific lectin-like	317	98.64	0
L_645_c0_g1_i1	putative lipid-transfer protein DIR1	1	C_12082_c0_g1_i1	putative lipid-transfer protein DIR1	108	97.09	0
L_50661_c0_g1_i1	oxygen-evolving enhancer protein 2, chloroplastic-like	1	C_14711_c0_g1_i1	oxygen-evolving enhancer protein 2, chloroplastic-like	133	97.74	0
L_16663_c0_g1_i2		1	C_20564_c0_g1_i1		642	89.54	2E-112
L_41624_c0_g1_i1	isocitrate lyase	1	C_15046_c0_g1_i1	isocitrate lyase	116	98.32	7E-180
L_33923_c0_g1_i1	galactinol synthase 2-like isoform X1	1	C_13705_c0_g1_i1	galactinol synthase 1-like	127	92.82	0
L_36400_c0_g1_i1	putative cell wall protein	0.98	C_26021_c0_g1_i1	putative cell wall protein	117	98.05	2E-98
L_53880_c0_g1_i1	uncharacterized protein LOC105056050	0.93	C_5177_c0_g1_i1	proactivator polypeptide-like 1	644	98.94	0
L_6399_c0_g1_i1	auxin-induced protein 22D-like	0.93	C_10469_c0_g1_i1	auxin-induced protein 22D-like	171	96.71	0
L_10569_c0_g2_i1		0.91	C_16356_c0_g1_i1		1072	93.62	0
L_21646_c1_g1_i3	protein HOTHEAD-like	0.91	C_14207_c0_g1_i1	protein HOTHEAD-like	330	96.12	0
L_22097_c0_g1_i1		0.82	C_9693_c0_g2_i1		155	92.67	0
L_30250_c0_g1_i1	polygalacturonase inhibitor	0.64	C_17486_c1_g1_i2	Polygalacturonase inhibitor	171	93.72	2E-170
L_50985_c0_g1_i1	putative phytosulfokines 6 isoform X1	0.47	C_22933_c0_g1_i1	putative phytosulfokines 6 isoform X2	136	95.71	0
L_39567_c0_g2_i1	profilin-1	0	C_15886_c0_g1_i1	profilin-1	615	97.97	0
L_5103_c0_g1_i1	trans-resveratrol di-O-methyltransferase-like	0	C_39904_c0_g1_i1	trans-resveratrol di-O-methyltransferase-like	430	79.15	0
L_24431_c0_g6_i1	60S ribosomal protein L24	0	C_1942_c0_g1_i1	60S ribosomal protein L24	278	97.76	0
L_3220_c0_g1_i1		0	C_1273_c0_g1_i1	chlorophyll a-b binding protein 6, chloroplastic	264	92.97	3E-47
L_16735_c0_g2_i2	uncharacterized protein LOC105047938	0	C_39063_c0_g1_i1	uncharacterized protein LOC105047938	172	92.49	0
L_256_c0_g1_i2	Os06g0133500	0	C_16734_c1_g2_i1	Os06g0133500	151	92.07	9E-165

Common genes with TPM value ≥50 between the two species, except when the TPM values of both genes is < 100. The genes were sorted based on the TPM value of *C. latifolia* along with the corresponding genes of *C. capitulata*. Note that there were no cases of genes that were highly expressed in both species. This pattern strongly suggests changes in the gene expression regulatory system due to divergence of two species

**Fig. 5:**
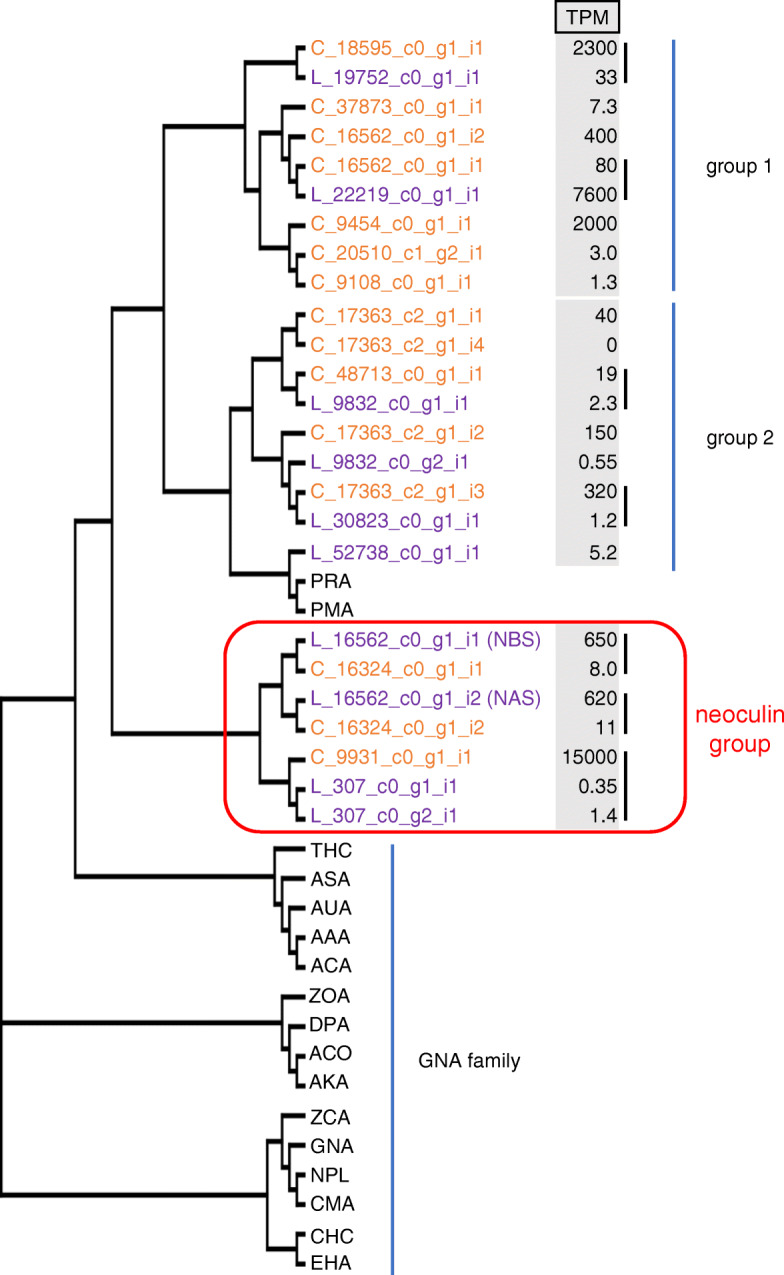
Phylogenetic analysis of neoculin-related transcripts uncovers the contrasting expression levels in the orthologs*.* Neoculin-related and GNA family members were aligned using ClustalX. The phylogenetic tree was constructed using the neighbor-joining method (bootstrap = 1000). De novo transcriptome transcript IDs for *C. latifolia* and *C. capitulata* are shown in purple and orange, respectively. L_16562_c0_g1_i1 and L_16562_c0_g1_i2 of *C. latifolia* correspond to NBS and NAS, respectively (see Additional file 7). Transcript per million (TPM) values are listed to the right of the transcript IDs. Transcripts from the two species encoding highly similar protein sequences are shown in pairs. Transcripts sharing high similarity with those of NBS and NAS are referred to as the neoculin group (indicated by the red frame). Groups of other highly similar predicted proteins are shown in groups 1 and 2. The vertical lines to the right of the TMP value indicate orthologous pairs in *C. latifolia* and *C. capitulata*. The sequences and species of origin of the selected GNA family members are as follows, with the structure name from the Protein Data Bank given in parentheses: ASA, *Allium sativum* (1BWU); GNA, *Galanthus nivalis* (1MSA); and NPL, *Narcissus pseudonarcissus* (1NPL). Other sequences were obtained from GenBank: PRA, *Polygonatum roseum* (AY899824); PMA, *Polygonatum multiflorum* (U44775); CMA, *Clivia miniata* (L16512); ZCA, *Zephyranthes candida* (AF527385); AAA, *Allium ascalonicum* (L12172); ACA, *Allium cepa* (AY376826); AUA, *Allium ursinum* (U68531); THC, *Tulipa* hybrid cultivar (U23043); ZOA, *Zingiber officinale* (AY657021); ACO, *Ananas comosus* (AY098512); AKA, *Amorphophallus konjac* (AY191004); DPA, *Dioscorea polystachya* (AB178475); CHC, *Cymbidium* hybrid cultivar (U02516); and EHA, *Epipactis helleborine* (U02515)

*neoculin-related transcripts (cf. Figure 5 and Additional file 6)

^a^Pident and *E*-value are BLASTN results performed with *C. latifolia* as query against *C. capitulata*

### Lectin genes expressed in *C. latifolia* and *C. capitulata* fruits

We previously demonstrated that *C. latifolia* fruits contain a taste-modifying protein consisting of a NBS-NAS heterodimer that is similar to lectins in the GNA family. We therefore investigated the number of *lectin* genes expressed in the fruits of *C. latifolia* and *C. capitulata* that were categorized into each of the 12 lectin families to better understand the general outline of the *GNA* gene family in these species. To determine the number of *lectin* genes, we performed tBLASTN searches against all transcripts in each species using the sequences of 12 representative lectins as query [41] (Table 4). In both species, the largest lectin family was the GNA family, which includes the *neoculin* (*NBS* and *NAS*) genes. Ten of the 45 *lectin* genes in *C. latifolia* and 13 of the 49 *lectin* genes in *C. capitulata* belonged to the *GNA* family. Thus, we analyzed the many *GNA* family genes in these species, including the *neoculin* genes, in more detail.

**Table 4:** Number of predicted *lectin* genes using tBLASTN in *C. latifolia* and *C. capitulata* fruits

Lectin domain	Model lectin	***C. latifolia***	***C. capitulata***
ABA domain	*Agaricus bisporus* agglutinin	0	0
Amaranthin domain	*Amaranthus caudatus* agglutinin	0	0
CRA domain	*Robinia pseudoacacia*chitinase-related agglutinin	3	4
Cyanovirin domain	*Nostoc ellipsosporum* agglutinin	0	0
EUL domain	*Euonymus europaeus* agglutinin	1	1
GNA domain	*Galanthus nivalis* agglutinin	10	13
Hevein domain	*Hevea brasiliensis* agglutinin	3	2
JRL domain	*Artocarus integer* agglutinin	9	4
Legume domain	*Glycine max* agglutinin	8	16
LysM domain	*Brassica juncea* LysM domain	1	1
Nictaba domain	*Nicotiana tabacum* agglutinin	10	8
Ricin-B domain	*Ricinus communis* agglutinin	0	0
Total number of *lectin* genes		45	49

### Analysis of GNA family and neoculin-related transcripts

We constructed a phylogenetic tree using the deduced protein sequences from 17 transcripts of well-known *GNA* family members and 25 full-length *neoculin*-related transcripts from *Curculigo* (10 from *C. latifolia* and 15 from *C. capitulata*; Fig. 5); the method used for sequence selection is shown in Additional file 5. The TPM values (calculated by RSEM) are listed after the transcript IDs. An alignment of all sequences is shown in Additional file 6. The *C. latifolia* transcript L_16562_c0_g1_i1 was a good match for NBS, while L_16562_c0_g1_i2 was a good match for NAS, except for one amino acid substitution (Additional file 7); these transcripts will be referred to as *NBS* and *NAS* hereafter. The predicted proteins derived from *neoculin*-related transcripts formed a distinct group separate from known GNA family members. Neoculin-like sequences formed one group that included NBS and NAS (named the ‘neoculin group’), as well as two other large groups (group 1 and group 2) (Fig. 5). In addition to NBS and NAS, the neoculin group also included proteins whose transcripts were highly expressed (C_9931_c0_g1_i1) and that presented the conserved amino acid residues critical for binding mannose (and thus have the potential for lectin activity). In addition, each transcript had an ortholog in both *Curculigo* species.

Many highly expressed transcripts belonged to group 1 (L_22219_c0_g1_i1 [TPM: 7600]; C_18595_c_g1_i1 [TPM: 2300]; C_9454_c0_g1_i1 [TPM: 2000]). Although these highly expressed transcripts encode proteins that are very similar to mannose-binding lectins, they are not mannose-binding lectins, as they lack the conserved and essential amino acid residues that form the mannose-binding sites. At this time, we do not know their physiological functions or the reason for their high expression. Predicted proteins encoded by group 2 transcripts were also relatively close to the lectins *Polygonatum multiflorum* agglutinin (PMA) and *Polygonatum roseum* agglutinin (PRA) from the *Polygonatum* genus. Unlike in group 1, there were no highly expressed transcripts in this group.

In each group, we detected *neoculin*-related orthologous transcripts with high similarity between *C. latifolia* and *C. capitulata*. The existence of many orthologs in each species, combined with the presence of relatively few common genes (comprising only approximately 40% of all transcripts in both species; Fig. 4), is noteworthy. We infer that these orthologs probably existed before the divergence of these two species, whereas their amino acid differences probably arose afterwards. Genetic diversity is beneficial for plants, including *Curculigo*, due to their lack of mobility to increase population survival against multiple stresses. It would be interesting to determine whether *Curculigo* plants other than *C. latifolia* and *C. capitulata* contain *neoculin*-related genes, especially genes in the *neoculin* group.

Within the *neoculin* group, we identified transcripts encoding proteins with high similarity to NBS and NAS in both *C. latifolia* and *C. capitulata*. Notably, although the corresponding *NBS* and *NAS* genes were highly expressed in *C. latifolia*, their *C. capitulata* orthologs were only weakly expressed (C_16324_c0_g1_i1 and C_16324_c0_g1_i2). The TPM values for *NBS* and *NAS* genes in *C. latifolia* were approximately the same, with 650 and 620 TPMs, respectively. This result is in agreement with the finding that their encoded proteins form a heterodimer [18]. Although C_9931_c0_g1_i1 was highly expressed in *C. capitulata*, with a TPM value of 15,000 (the fifth highest expression level among all *C. capitulata* transcripts), its *C. latifolia* ortholog (L_307_c0_g1_i1 and L_307_c0_g2_i1) was expressed at a very low level. In order to verify the results of RNA-seq, qRT-PCR analyses for the genes of the *neoculin* group in two species were performed (Additional files 8 and 9). Then, we compared the expression levels using a *ubiquitin* gene of each species as a reference gene. In *C. latifolia*, the expression levels of *NBS* and *NAS* were almost same, and that of L_307_c0_g1_i1 and L_307_c0_g2_i1 was considerably lower than them. In *C. capitulata*, the expression levels of C_16324_i1 and C_16324_c0_g1_i2 were very small, and that of C_9931_c0_g1_i1 was very large. These results support TPM values estimated from RNA-seq analysis. In addition, comparing the high-low relationship of the expression level in two species, results obtained by RNA-seq analysis was also supported by qRT-PCR analyses. Curiously, in all three groups (neoculin group, groups 1 and 2) for which there were orthologs in both species, if a gene was highly expressed in one species, its ortholog was weakly expressed in the other species; we did not identify a single case where orthologs were highly expressed in both species. The data shown in Table 3 also support this pattern. These results strongly suggest changes in the gene expression regulatory system due to divergence of the two species.

**Fig. 6:**
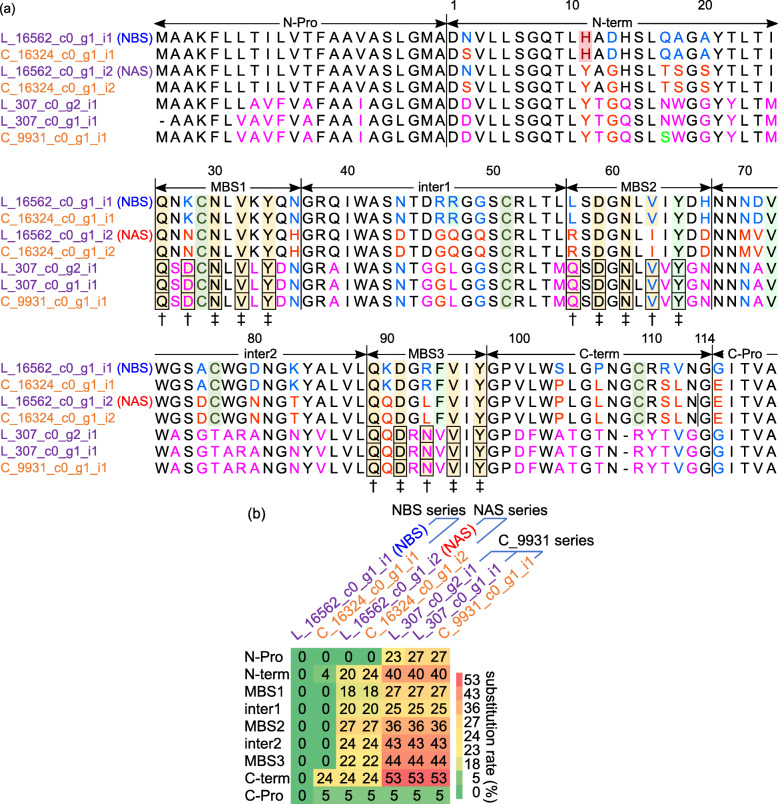
The essential amino acid residues in neoculin group members have been conserved. **a** Amino acid sequence alignment of neoculin group members from *C. latifolia* and *C. capitulata* fruits*.* In each alignment, the residues that are shared with only NBS or only NAS are shown in blue and red, respectively. The residues that are not consistent with NBS or NAS are shown in pink, and those that are consistent with only C_9931_c0_g1_i1 (Ser17) are shown in light green. His-11 and Cys residues are highlighted in dark red and dark green, respectively. Arg-48, Tyr-65, Val-72, and Phe-94 are highlighted in pale green. Mannose-binding sites (MBS, QxDxNxVxY) are indicated by a dagger (†), and conserved residues are highlighted in yellow. MBS residues that are conserved in all sequences are indicated by a double dagger (‡). MBS residues in L_307_c0_g2_i1, L_307_c0_g1_i1, and C_9931_c0_g1_i1 are shown in boxes. The predicted proteins were divided into nine regions—N-Pro, N-term, MBS1, inter1, MBS2, inter2, MBS3, C-term, and C-Pro—based on the regions removed after signal-peptide cleavage, the N- or C-terminal regions, the regions of MBS 1 to 3, and the regions between the MBSs. **b** Amino acid residue substitutions in proteins from the neoculin group. The region from inter2 to C-term is the primary region of sequence diversity in the neoculin group. The values shown in the heatmap are amino acid substitution rates (%) of neoculin group. The NBA sequence was used as the reference

Next, we aligned the deduced amino acid sequences for the proteins belonging to the neoculin group (Fig. 6a). We divided the sequences into nine regions, including the regions removed by cleavage of the secretion signal peptide and three mannose binding site (MBS)-like regions: N pro-sequence (N-Pro), N-terminal (N-term), MBS1, inter1, MBS2, inter2, MBS3, C-terminal (C-term), and C pro-sequence (C-Pro). The His-11 residue was present in the N-term region of NBS and in the predicted proteins encoded by transcripts L_16562_c0_g1_i1 in *C. latifolia* and C_16324_c0_g1_i1 in *C. capitulata*. This site essential for the pH-dependent taste-modifying activity of neoculin. By contrast, transcripts C_9931_c0_g1_i1 in *C. capitulata* and L_307_c0_g1_i1 and L_307_c0_g2_i1 in *C. latifolia* (abbreviated ‘C_9931 series’) did not code for His-11, which was replaced by Tyr-11, as in NAS. In addition, Cys-77 and Cys-109, which form an intermolecular disulfide bond between NBS and NAS, were present within the inter2 and C-term regions in both species, but were absent in the C_9931 series. Thus, it is likely that proteins corresponding to the C_9931 series do not form dimers.

Four residues are responsible for the binding and activation of the human sweet receptor: Arg-48, Tyr-65, Val-72, and Phe-94 [26]. Although Tyr-65 and Val-72 were identified in the C_9931 series, Leu-48 and Val-94 were missing. The lack of His-11 and these four indispensable residues, as well as the lack of dimerization, indicate that the C_9931 series proteins may not possess the sweet taste or taste-modifying properties of classic neoculin. Indeed, a preliminary test indicated that *C. capitulata* fruits did not have a sweet taste or taste-modifying properties despite the high expression level of C_9931_c0_g1_i1 (data not shown). Three sites similar to the MBS were present in the MBS1, MBS2, and MBS3 regions of this protein. Moreover, whereas NBS and NAS lack the essential residues of the MBS, all of these residues were conserved in C_9931_c0_g1_i1, making C_9931_c0_g1_i1 a likely lectin candidate.

Based on this protein alignment, we investigated all amino acid substitutions in each region in comparison to the two reference sequences, NBS and NAS (Additional file 10). The amino acid substitution rate with reference to NBS is shown in the heatmap in Fig. 6b. Between the NBS series and the NAS series, 18 to 27% of substitutions occurred in the overall regions from the N-term region to C-term region (23%, 26 of 114 residues in NBS). The highest substitution rate was 27% in the MBS2 region, followed by 24% in the inter2 and C-term regions. In the C_9931 series, the highest substitution rate was 53% in the C-term region, followed by the MBS3 region (44%) and inter2 region (43%). These results suggest that the region from inter2 to C-term is the main source of sequence diversity among neoculin group members.

### Biochemical analysis

**Fig. 7:**
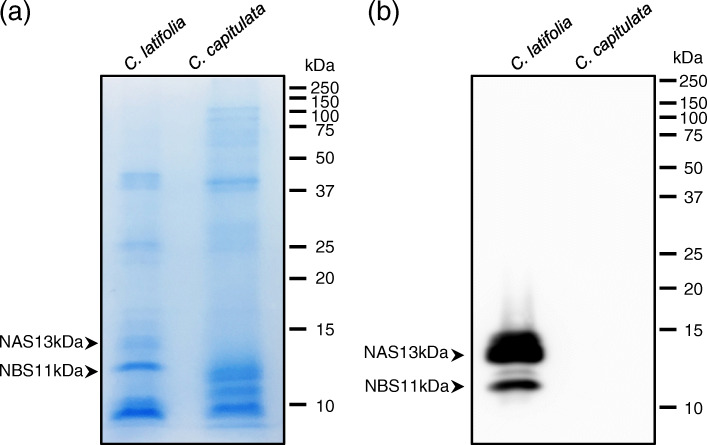
Biochemical analysis of *C. latifolia* and *C. capitulata* fruits suggests only *C. latifolia* possesses neoculin*.* Extracts from one fruit each of *C. latifolia* and *C. capitulata* were subjected to SDS-PAGE. 20 μg protein of each fruit extract was applied to each well. **a** CBB staining. **b** Immunoblotting

We extracted proteins from *C. latifolia* and *C. capitulata* fruits and subjected them to SDS-PAGE, followed by Coomassie brilliant blue (CBB) staining and immunoblotting using a mixture of polyclonal anti-NAS and anti-NBS specific antibodies (Fig. 7 and Additional file 11). The CBB-stained gel is shown in Fig. 7a and the corresponding immunoblot in Fig. 7b. By CBB staining, we detected an 11-kDa band representing NBS and a 13-kDa band representing NAS in *C. latifolia* fruit samples (Fig. 7a). In *C. capitulata* fruits, some bands around 11 kDa may be the protein encoded by C_9931_c0_g1_i1, which had a high TPM value. Immunoblotting confirmed the identity of the bands corresponding to NBS and NAS in *C. latifolia* fruits. However, we detected no such bands in *C. capitulata* fruits (Fig. 7b), perhaps because NBS and NAS accumulate at very low levels in this species, as reflected by the low TPM values of their encoding transcripts (as described above). The amino acid sequence of the C-term region, which is recognized by the antibody, was also very different in C_9931_c0_g1_i1 compared to both NBS and NAS, which is consistent with the finding that the proteins detected by CBB staining were not detected by immunoblotting.

## Discussion

The *C. latifolia* and *C. capitulata* transcriptomes contain many *neoculin*-related genes that are similar within and between species. This diversity is thought to result from gene duplication, which is known to contribute to plant evolution [41–47]. Such gene duplication might place some genes under the same transcriptional regulation. The *neoculin* genes *NBS* and *NAS* are likely paralogs that arose due to tandem duplication before the divergence of *C. latifolia* and *C. capitulata*. The characteristics of *NBS* and *NAS* genes in *C. latifolia* and *C. capitulata* are summarized in Table 5. Both *C. latifolia* and *C. capitulata* produce *NBS* and *NAS* transcripts, and the sequences of the C_9931 series transcripts matched those of active *GNA* family members. However, their expression levels in the two species were very different.

**Table 5:** Summary of *neoculin* group transcripts in fruits of two *Curculigo* species

	Transcript ID	Reference transcript	No. of substitutions (amino acid)	Heterodimerization (no. of Cys)	Lectin activity (no. of MBS^**a**^)	Taste modification	Expression (TPM^b^)
***C. latifolia***	L_16562_c0_g1_i1 (NBS)	*NBS*	0	Yes (4)	No (0)	Yes	High (650)
	L_16562_c0_g1_i2 (NAS)	*NAS*	1	Yes (4)	No (0)	Yes	High (620)
	L_307_c0_g2_i1	C_9931_c0_g1_i1	1	Unknown (2)	Unknown (3)	Unknown	Very low (1.4)
	L_307_c0_g1_i1	C_9931_c0_g1_i1	1	Unknown (2)	Unknown (3)	Unknown	Very low (0.35)
***C. capitulata***	C_16324_c0_g1_i1	*NBS*	6	Probably Yes (4)	Probably No (0)	Probably Yes	Low (8.0)
	C_16324_c0_g1_i2	*NAS*	0	Probably Yes (4)	Probably No (0)	Probably Yes	low (11)
	C_9931_c0_g1_i1	C_9931_c0_g1_i1	–	Unknown (2)	Unknown(3)	Unknown (Fruits have no activity)	Very high (15,000)

The number of amino acid residues difference from the reference sequences, the potential for heterodimerization, lectin activity, taste modification, and expression levels of the transcripts from *C. latifolia* or *C. capitulata* fruits are summarized. As the reference sequences, amino acid sequences of NBS, NAS, and C_9931_c0_g1_i1 of *C. capitulata* were used

^**a**^*MBS* mannose-binding site

^b^*TPM* transcripts per million

*C. latifolia* fruits have been reported to accumulate 1.3 mg neoculin g^− 1^ fresh pulp. Because neoculin is 550 times as sweet as sucrose [19, 20], one gram of *C. latifolia* fruit pulp is thus estimated to be equivalent to 715 mg of sucrose in sweetness, explaining the sweet taste of these fruits. Given that the TPM values of the *neoculin* genes in *C. capitulata* were only 1/60 those detected in *C. latifolia*, *C. capitulata* fruits would be expected to contain only approximately 22 μg neoculin g^− 1^ fresh pulp and have the same sweetness as 12 mg of sucrose. Based on these values, it seemed likely that *C. capitulata* fruits would not taste sweet, which we confirmed in a preliminary test. Thus, neoculin levels, and therefore taste, differ greatly between these fruits, paralleling the difference in the expression of *NBS* and *NAS* genes in the two species. The taste of *C. latifolia* fruits may strongly influence its survival strategies. For example*,* the sweet taste conferred by neoculin may facilitate seed spread by animals.

The structure of the taste-modifying protein miraculin is similar to those of the soybean (*Glycine max*) Kunitz trypsin inhibitor and thaumatin, a sweet protein with an α-amylase or trypsin-inhibitor-like structure. Similarly, neoculin has a structure similar to that of lectin, a common molecular structure in plants [48–54]. Trypsin inhibitors, amylase inhibitors, and lectins commonly accumulate in fruits and seeds. The diversity of these proteins arose from gene duplications and mutations during evolution. It appears that over the course of evolution, neoculin, miraculin, and thaumatin all acquired sweetness or taste-modifying activity in regard to human senses.

Lectins are thought to play important protective and storage roles in general plants. Thus, the high expression levels of *lectin* genes in *C. capitulata* fruits is likely to reflect important roles of lectins in this plant. In contrast, the low expression levels of *neoculin* genes in *C. capitulata* suggest that the encoded protein may be less beneficial in this species. Similarly, and in contrast to *C. capitulata*, active *GNA* family members were barely expressed in *C. latifolia* fruits. *Neoculin* genes were highly expressed in *C. latifolia* but weakly expressed in *C. capitulata* despite the similar vegetative appearance of the two plants (Fig. 1). These physiological differences might be due to mutation(s) of the *cis*-regulatory elements in these genes. *Cis*-elements, including promoters, enhancers, and silencers, are very important for the regulation of gene expression [41, 55–58]. Likewise, the different expression levels of related genes in *C. latifolia* vs. *C. capitulata* might be caused by mutations in their *cis*-elements. For example, the *cis*-elements of the *NBS* and *NAS* genes may have mutated after the divergence of the two species, or the genes may have acquired mutations or lost *cis*-elements during the gene duplication events that led to their divergence, leading to different expression patterns. Deciphering the genomic information of these two species further might help verify this notion and distinguish among these possible mechanisms.

## Conclusions

RNA-seq analysis and de novo transcriptome assembly of *C. latifolia* and *C. capitulata* fruits revealed the presence of numerous *neoculin*-like genes. Among the various *neoculin*-related genes that arose from gene duplication, several mutations accumulated, resulting in the genes encoding NBS and NAS. These proteins form the heterodimeric protein neoculin, which exhibits taste-modifying activity in humans. Our comprehensive investigation of the genes expressed in the fruits of these two *Curculigo* species will help uncover the origin of neoculin at the molecular level.

## Methods

### Plant materials

*C. latifolia* (voucher ID 26092) was obtained from the Research Center for Medicinal Plant Resources, National Institutes of Biomedical Innovation, Health, and Nutrition, Tsukuba, Japan (originated in Indonesia). *C. capitulata* (voucher ID 31481) was obtained from The Naito Museum of Pharmaceutical Science and Industry, Kakamigahara, Japan. The plants were cultivated in a greenhouse at the Yamashina Botanical Research Institute. Photographs of the fruits of these plants are shown in Fig. 1.

### Fruit setting

*C. latifolia* flowers were pollinated by hand in the morning on the first day of flowering. *C. capitulata* flowers were placed in 50 ppm of 1-naphthylacetic acid (NAA) in the morning of both the first and second days of flowering. This is the first report of a method to induce *C. capitulata* fruit set through plant hormone application. About 60 days after flowering, mature fruits were harvested and immediately soaked in RNA later™ solution (Thermo Fisher Scientific, MA. USA). The fruits were stored at − 80 °C until use. The samples were ground into a powder in liquid nitrogen prior to RNA extraction. Total RNA was extracted from the frozen samples using the phenol-SDS method, and poly(A)^+^ mRNA was purified using an mRNA Purification Kit (Amersham Biosciences, Buckinghamshire, UK).

### Sequencing

mRNA sequencing was performed by Hokkaido System Science Co., Ltd. (Hokkaido, Japan). A cDNA library was generated using TruSeq RNA Sample Prep Kit v2 (Illumina, Inc., CA. USA) and sequenced on an Illumina HiSeq 2500 platform (101 bp read length, paired-end, unstranded). The raw reads were cleaned using cutadapt1.1 [59] and trimmomatic0.32 [60]. We removed adapter sequences, low-quality sequences (reads with ambiguous ‘N’ bases), and reads with *Q*-value < 20 bases. Sequences smaller than 50 bases were eliminated. The remaining high-quality reads were assembled into contigs using Trinity2.11 [61] with default options. We quantified transcript levels as transcripts per million (TPM) values using Bowtie1.12 [62] and RSEM (RNA-Seq by Expectation-Maximization) [63] in the Trinity package.

### Sequence clustering

The assembled sequences were compared against the NCBI NR, prot-plant from RefSeq, UniProt, the rice genome (Os-Nipponbare-Reference-IRGSP-1.0, Assembly: GCF_001433935.1), and the Arabidopsis genome (*Arabidopsis thaliana*, Assembly: GCF_000001735.4) with an *E*-value <1e^− 10^. BLAST analysis was performed using BLAST version 2.2.31. CD-Hit (cd-hit-est) [64, 65] was used for clustering with the option of threshold (−c) 0.9 to obtain unigenes.

### Comparison of gene expression in *C. latifolia* vs. *C. capitulata* fruits

To compare the transcripts in *C. latifolia* vs. *C. capitulata* fruits*,* a BLASTN search was performed with *E*-value <1e^− 5^ using each transcript from one species as the query against all transcripts from the other species, and then the best hits were selected. cDNA was synthesized from 1 μg of total RNA using SuperScript IV Reverse Transcriptase (Thermo Fisher Scientific, MA. USA) according to the manufacturer’s instructions. PowerUp SYBR Green Master Mix (Thermo Fisher Scientific, MA. USA) was used with an ABI 7500 real-time PCR system (Thermo Fisher Scientific, MA. USA). The thermal cycling program was performed using the following parameters: denaturation at 95 °C for 2 min, prior to 40 amplification cycles (95 °C for 15 s, 60 °C for 1 min). Melting curves were constructed after 40 cycles to confirm the specificity of the reactions. The 2^-ΔΔCT^ method was used to calculate the relative expression of six genes following normalization to L_19431_c0_g1_i2 for *C. latifolia* and C_20039_c0_g6_i1 for *C. capitulata*, which are probably *ubiquitin* genes in *C. latifolia* and *C. capitulata.* The primer sequences are shown in Additional file 8.

### Identification of *lectin* gene transcripts in *C. latifolia* and *C. capitulata* fruits

A tBLASTN search (*E*-value <1e^− 4^; other options set to the default) was performed against all transcripts in *C. latifolia* and *C. capitulata* fruits with the following protein sequences as the queries, which represent each plant lectin family [41]: *Agaricus bisporus* (white mushroom) agglutinin (UniProtKB/Swiss-Prot: Q00022.3—ABA), *Amaranthus caudatus* (foxtail amaranth) agglutinin (GenBank: AAL05954.1—amaranthin), *Robinia pseudoacacia* (black locust) chitinase-related agglutinin (GenBank: ABL98074.1—CRA), *Nostoc ellipsosporum* (cyanobacterium) agglutinin (UniProtKB/Swiss-Prot: P81180.2—cyanovirin), *Euonymus europaeus* (European spindle) agglutinin (GenBank: ABW73993.1—EUL), *Galanthus nivalis* (snowdrop) agglutinin (UniProtKB/Swiss-Prot: P30617.1—GNA), *Hevea brasiliensis* (rubber tree) agglutinin (GenBank: ABW34946.1—hevein), *Artocarpus integer* (chempedak) agglutinin (GenBank: AAA32680.1—JRL), *Glycine max* (soybean) agglutinin (UniProtKB/Swiss-Prot: P05046.1—legume lectin), *Brassica juncea* (brown mustard) LysM domain (GenBank: BAN83772.1—LysM), *Nicotiana tabacum* (tobacco) agglutinin (GenBank: AAK84134.1—Nictaba), and the lectin chain of *Ricinus communis* (castor bean) agglutinin (GenBank: PDB: 2AAI_B—ricin B). The top hits were selected.

### Phylogenetic analysis of the GNA protein family

The sequences of 17 well-known GNA proteins were selected according to Shimizu-Ibuka et al. [36]. The protein sequences for ASA, *Allium sativum* (garlic) (1BWU); GNA, *Galanthus nivalis* (snowdrop) (1MSA); and NPL, and *Narcissus pseudonarcissus* (wild daffodil) (1NPL) were obtained from the Protein Data Bank. Others sequences were selected from GenBank as follows: PRA, *Polygonatum roseum* (AY899824); PMA, *Polygonatum multiflorum* (Solomon’s seal) (U44775); CMA, *Clivia miniata* (kaffir lily) (L16512); ZCA, *Zephyranthes candida* (autumn zephyr lily) (AF527385); AAA, *Allium ascalonicum* (shallot) (L12172); ACA, *Allium cepa* (onion) (AY376826); AUA, *Allium ursinum* (wild garlic) (U68531); THC, *Tulipa* hybrid cultivar (tulip) (U23043); ZOA, *Zingiber officinale* (ginger) (AY657021); ACO, *Ananas comosus* (pineapple) (AY098512); AKA, *Amorphophallus konjac* (konjac) (AY191004); DPA, *Dioscorea polystachya* (yam tuber) (AB178475); CHC, *Cymbidium* hybrid cultivar (cymbidium) (U02516); and EHA, *Epipactis helleborine* (broad-leaved helleborine) (U02515). These 17 sequences and 25 neoculin-related proteins predicted from full-length transcripts (10 transcripts from *C. latifolia* and 15 from *C. capitulata*; Fig. 5) were aligned using ClustalX [66], and the neighbor-joining tree was generated and analyzed with 1000 replicates for bootstrap testing. A complete list of sequences used is given in additional file 12.

### Biochemical analysis

SDS-PAGE was carried out using fruit extracts from *C. latifolia* and *C. capitulata*. The proteins were visualized by Coomassie brilliant blue (CBB) staining. Immunoblot analysis was carried out using anti-NBS and anti-NAS specific polyclonal antibodies [38, 67], which were raised against the C terminus of NAS or NBS, respectively. Preparation and purification of fruit extracts were performed as described previously [18, 38]. Each 0.1 g pulp sample was treated with 0.5 mL of 0.5 M NaCl to obtain an extract, which was combined with the appropriate volume of buffer containing 2-mercaptoethanol for SDS-PAGE. After the SDS-PAGE, proteins were transferred to PVDF membrane pore size of 0.45 μm (Merck Millipore, MA. USA). The membrane was soaked in Tris-buffered saline/Tween-20 (TBST) containing 5% skim-milk to block the non-specific protein reaction. After blocking, the membrane was reacted with the mixture of anti-NBS and anti-NAS specific polyclonal antibodies diluted 1:500 in TBST solution for 1 h at room temperature. And then, the membrane was washed with TBST solution at three times for 5 min. Next, the membrane was reacted with Rabbit IgG HRP Linked Whole Ab (Sigma-Aldrich, MO. USA) diluted 1:4000 in TBST solution for 1 h at room temperature. The membrane was washed with TBST solution at three times for 5 min. Signals were visualized with Clarity Western ECL Substrate kit (BIO-RAD, CA. USA) according to the protocol attached to ECL Kit. The signals were detected at 428 nm for 20 s exposure using Luminescent Image Analyzer (Image Quant LAS 4000 mini, GE Healthcare, IL. USA).

## Supplementary Information

**Additional file 1: Supplemental Figure 1**. Length distribution of the assembled transcripts (> 1 TPM) from *C. latifolia* (purple) and *C. capitulata* (orange) fruits

**Additional file 2: Supplemental Figure 2**. Distribution of transcripts per million (TPM) values of the assembled transcripts from *C. latifolia* (purple) and *C. capitulata* (orange) fruits. Average, median and mode of *C. latifolia* were 11.7, 1.6, and 2, respectively, and those of *C. capitulata*, 13.0, 1.9, and 2, respectively

**Additional file 3: Supplemental annotation**. Transcripts from *C. latifolia* and *C. capitulata* fruits were annotated by BLASTX (*E*-value <1e^− 10^) against the NCBI NR, RefSeq, UniProt, and COG databases and genomes from rice (Os-Nipponbare-Reference-IRGSP-1.0, Assembly: GCF_001433935.1) and Arabidopsis (*Arabidopsis thaliana*, Assembly: GCF_000001735.4). Percentage identity (Pident) and *E*-value are BLASTN results performed using *C. latifolia* as the query against *C. capitulata*. Expression values (TPM), unigenes clustered by CD-Hit, and the unique or common genes from *C. capitulata* or *C. latifolia* are also included

**Additional file 4: Supplemental Figure 3**. Gene Ontology (GO) annotation of transcripts from *C. latifolia* (purple) and *C. capitulata* (orange) fruits. In total, 28,100 (*C. latifolia*) and 29,614 (*C. capitulata*) transcripts were classified based on GO terms. No significant differences were observed between the two species

**Additional file 5: Supplemental Table 1**. Selection of sequences for phylogenetic analysis using BLAST search of transcripts from *C. latifolia* and *C. capitulata* fruits*.* The query sequences were the amino acid sequences (AA) of GNA (UniProtKB/Swiss-Prot: P30617.1) and the nucleotide sequences (nucl) and AA number of NBS (GenBank: X64110.1, GenBank: CAA45476.1) and NAS (GenBank: AB167079.1, GenBank: BAD29946.1). Each top hit was selected. The contigs that were selected for each query and used as neoculin-related sequences in phylogenetic analysis (Fig. 5) are indicated by checkmarks (✓)

**Additional file 6: Supplemental Figure 4**. Amino acid sequence alignment of 10 *C. latifolia* transcripts, 15 *C. capitulata* transcripts, and 17 well-known GNA family members used in the phylogenetic analysis shown in Fig. 5

**Additional file 7: Supplemental Figure 5**. Comparison of the protein sequences of neoculin (NBS and NAS) from public databases and the *Curculigo latifolia* proteins identified in the present study. Amino acid residues that differ between NBS and NAS are shown in blue for NBS residues and red for NAS residues. Asn-2 of L_16562_c0_g1_i2 was the only residue that differed with NAS, which has Ser at this position (GenBank: BAD29946.1). These de novo assemblies were good matches with the known sequences (NBS and NAS). This level of matching supports the accuracy of the assembly

**Additional file 8: Supplemental Table 2**. Primer information used for qRT-PCR. qRT-PCR analyses were performed on the *neoculin* related genes from *C. latifolia* and *C. capitulata* fruits

**Additional file 9: Supplemental Table 3**. Relative quantification of the *neoculin* related genes from *C. latifolia* and *C. capitulata* fruits by qRT-PCR. The mean values of qRT-PCR from three independent biological replicates were normalized to *ubiquitin* gene of each species

**Additional file 10: Supplemental Table 4**. Numbers of amino acid substitutions in neoculin group proteins. The values show the number of substituted amino-acids in each region. The predicted proteins were divided into nine regions—N-Pro, N-term, MBS1, inter1, MBS2, inter2, MBS3, C-term and C-Pro—based on the regions removed by processing, the N- or C-terminal regions, the mannose-binding sites MBS 1 to 3, and the regions between the MBSs. “A” indicates residues in NAS that are different from those of NBS. “B” indicates residues in NBS that are different from those of NAS. “C” indicates residues different from both NBS and NAS. “D” indicates the residues only present in C_9931_c0_g1_i1

**Additional file 11: Supplemental Figure 6**. Original pictures of Fig. 7. (a) CBB staining gel. (b) PVDF membrane after reaction under bright field. (c) Immunoblotting membrane reacted with ECL. The signals were detected at 428 nm with the exposure time of 20 s. (d) Overlay image of (b) and (c)

**Additional file 12: Supplemental Table 5**. Accession number of sequences obtained from web-based sources

## Data Availability

The raw data and processed data from this study have been uploaded to the NCBI Gene Expression Omnibus (GSE151377) and are available in the NCBI database under accession number PRJNA635640, https://www.ncbi.nlm.nih.gov/bioproject/635640. NCBI NR (https://ftp.ncbi.nih.gov/blast/db/FASTA/nr.gz), prot-plant from RefSeq (https://ftp.ncbi.nlm.nih.gov/refseq/release/plant/), and UniProt (https://ftp.uniprot.org/pub/databases/uniprot/knowledgebase/) databases were used in this study. Accession numbers of sequences are given in Additional file 12.

## Abbreviations

AAA: *Allium ascalonicum* agglutinin
ACA: *Allium cepa* agglutinin
ACO: *Ananas comosus* lectin
AKA: *Amorphophallus konjac* agglutinin
ASA: *Allium sativum* agglutinin
AUA: *Allium ursinum* agglutinin
CHC: *Cymbidium* hybrid cultivar agglutinin
CMA: *Clivia miniata* agglutinin
COG: Cluster of Orthologous Groups
DPA: *Dioscorea polystachya* agglutinin
ECL: enhanced chemiluminescence
EHA: *Epipactis helleborine* agglutinin
GNA: *Galanthus nivalis* agglutinin
GO: Gene Ontology
HRP: Horseradish peroxidase
NAA: 1-naphthylacetic acid
NAS: Neoculin acidic subunit
NCBI: The National Center for Biotechnology Information
NBS: Neoculin basic subunit
NGS: Next generation sequencing
NPL: *Narcissus pseudonarcissus* lectin
PMA: *Polygonatum multiflorum* agglutinin
PRA: *Polygonatum roseum* agglutinin
PVDF: Polyvinylidene difluoride
TBST: Tris-buffered saline/Tween-20
THC: *Tulipa* hybrid cultivar lectin
ZOA: *Zingiber officinale* agglutinin
TPM: Transcripts per million
ZCA: *Zephyranthes candida* agglutinin
