# Supplementary material for: De novo transcriptome analysis and comparative expression profiling of genes associated with the taste-modifying protein neoculin in Curculigo latifolia and Curculigo capitulata fruits
Source: BMC Genomics. 2021 May 13;22:347. doi: 10.1186/s12864-021-07674-3 (PMC8120819; doi:10.1186/s12864-021-07674-3)
Supplement: Supplementary file 1 — Additional file 1: Supplemental Figure 1. Length distribution of the assembled transcripts (> 1 TPM) from C. latifolia (purple) and C. capitulata (orange) fruits [file 12864_2021_7674_MOESM1_ESM.pdf]

Additional File 1. Supplemental Figure 1.

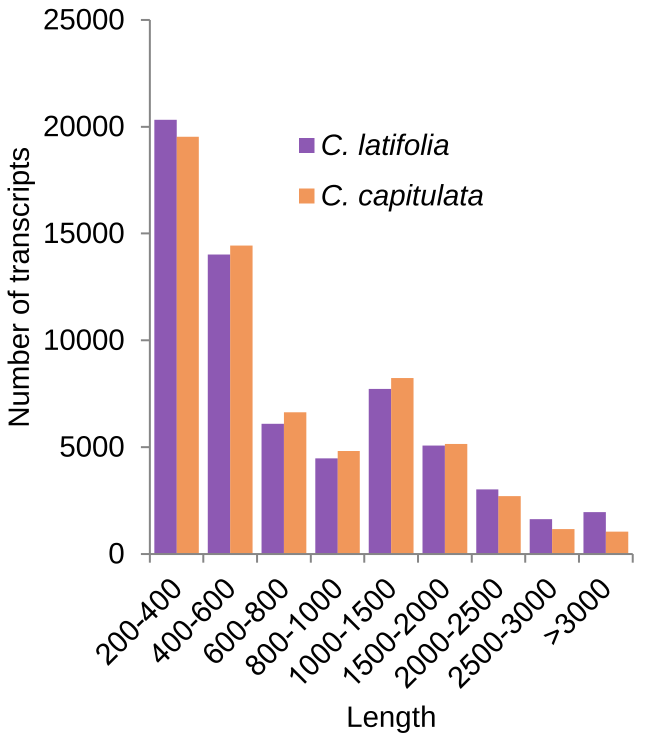

Length distribution of the assembled transcripts (>1 TPM) from *C. latifolia* (purple) and *C. capitulata* (orange) fruits.
