# Supplementary material for: De novo transcriptome analysis and comparative expression profiling of genes associated with the taste-modifying protein neoculin in Curculigo latifolia and Curculigo capitulata fruits
Source: BMC Genomics. 2021 May 13;22:347. doi: 10.1186/s12864-021-07674-3 (PMC8120819; doi:10.1186/s12864-021-07674-3)
Supplement: Supplementary file 2 — Additional file 2: Supplemental Figure 2. Distribution of transcripts per million (TPM) values of the assembled transcripts from C. latifolia (purple) and C. capitulata (orange) fruits. Average, median and mode of C. latifolia were 11.7, 1.6, and 2, respectively, and those of C. capitulata, 13.0, 1.9, and 2, respectively [file 12864_2021_7674_MOESM2_ESM.pdf]

Additional File 2. Supplemental Figure 2.

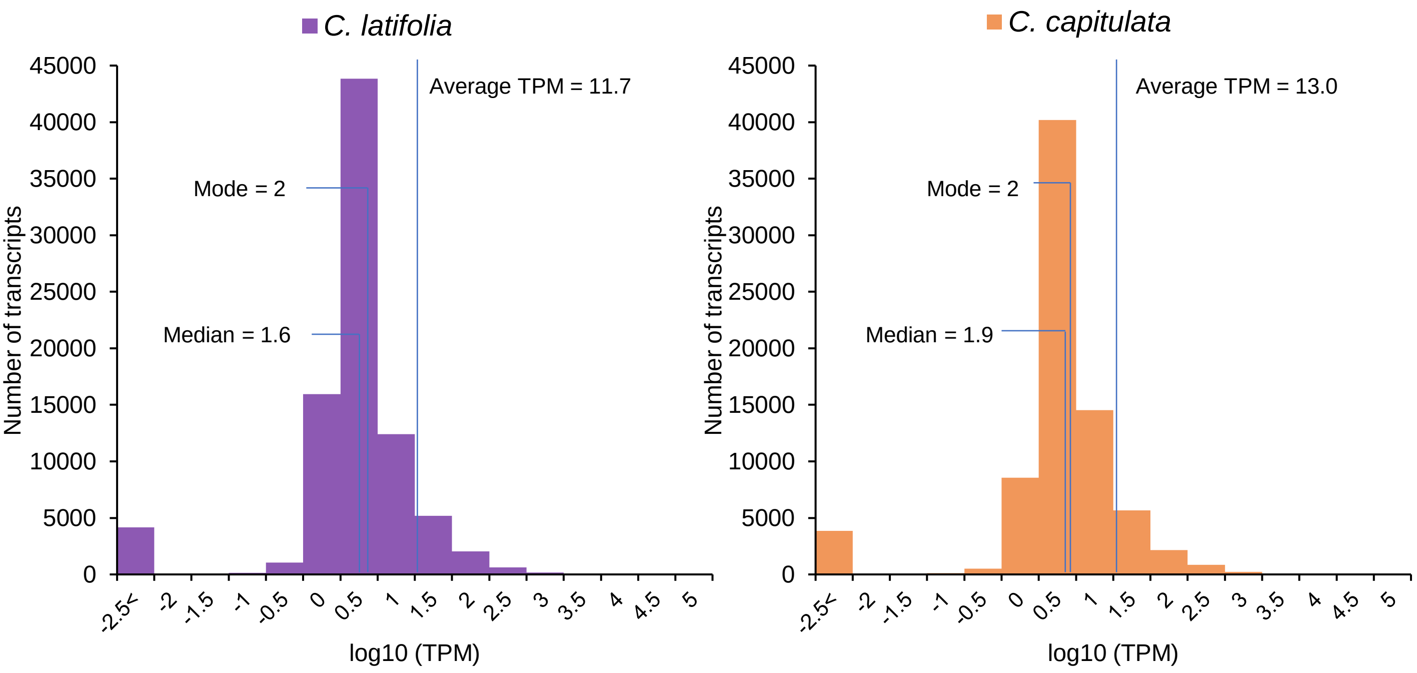

Distribution of transcripts per million (TPM) values of the assembled transcripts from *C. latifolia* (purple) and *C. capitulata* (orange) fruits.
Average, median and mode of *C. latifolia* were 11.7, 1.6, and 2, respectively, and those of *C. capitulata*, 13.0, 1.9, and 2, respectively.
