# Supplementary material for: De novo transcriptome analysis and comparative expression profiling of genes associated with the taste-modifying protein neoculin in Curculigo latifolia and Curculigo capitulata fruits
Source: BMC Genomics. 2021 May 13;22:347. doi: 10.1186/s12864-021-07674-3 (PMC8120819; doi:10.1186/s12864-021-07674-3)
Supplement: Supplementary file 4 — Additional file 4: Supplemental Figure 3. Gene Ontology (GO) annotation of transcripts from C. latifolia (purple) and C. capitulata (orange) fruits. In total, 28,100 (C. latifolia) and 29,614 (C. capitulata) transcripts were classified based on GO terms. No significant differences were observed between the two species [file 12864_2021_7674_MOESM4_ESM.pdf]

Additional File 4. Supplemental Figure 3.

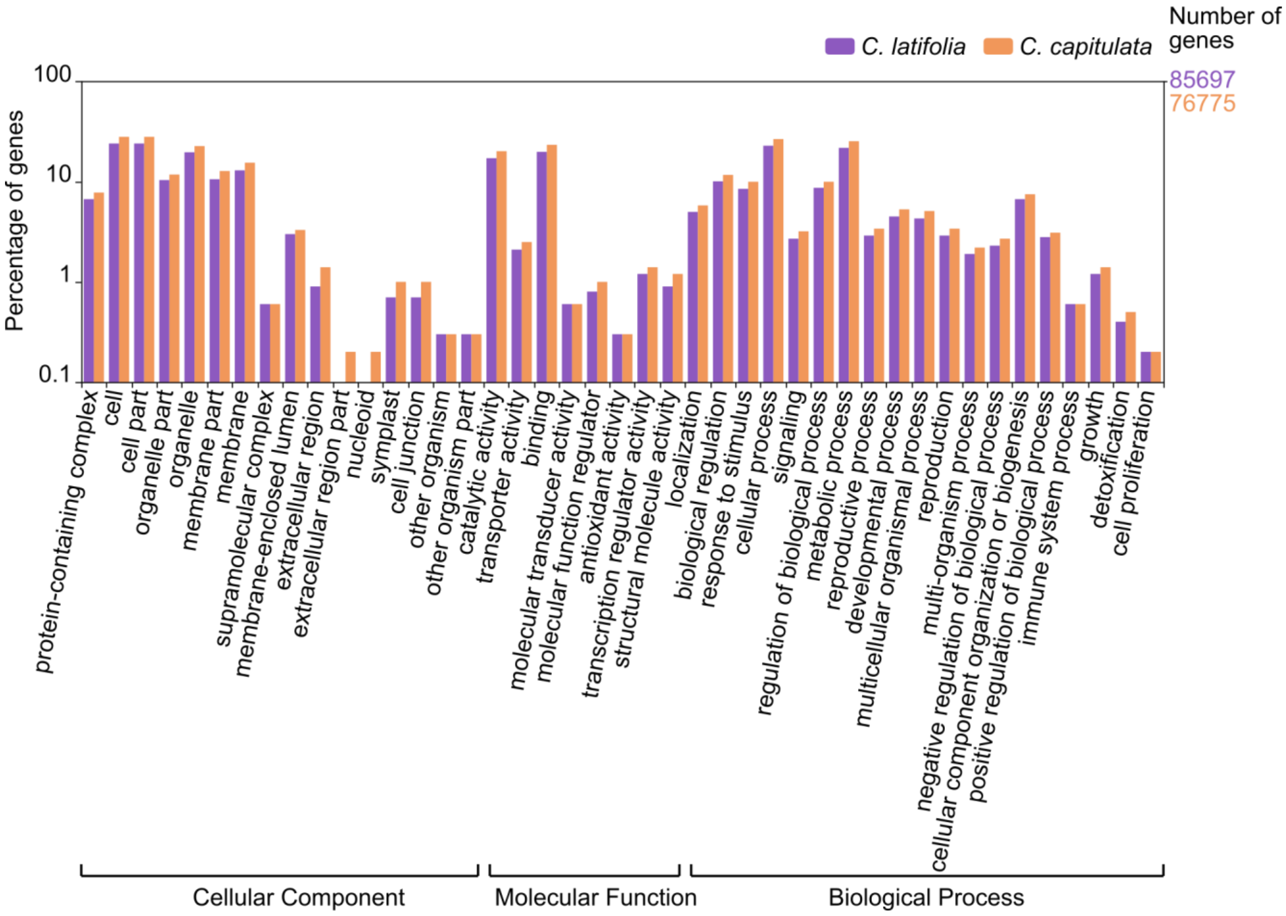

Gene Ontology (GO) annotation of transcripts from *C. latifolia* (purple) and *C. capitulata* (orange) fruits.

In total, 28,100 (*C. latifolia*) and 29,614 (*C. capitulata*) transcripts were classified based on GO terms. No significant differences were observed between the two species.
