# Supplementary material for: De novo transcriptome analysis and comparative expression profiling of genes associated with the taste-modifying protein neoculin in Curculigo latifolia and Curculigo capitulata fruits
Source: BMC Genomics. 2021 May 13;22:347. doi: 10.1186/s12864-021-07674-3 (PMC8120819; doi:10.1186/s12864-021-07674-3)
Supplement: Supplementary file 11 — Additional file 11: Supplemental Figure 6. Original pictures of Fig. 7. (a) CBB staining gel. (b) PVDF membrane after reaction under bright field. (c) Immunoblotting membrane reacted with ECL. The signals were detected at 428 nm with the exposure time of 20 s. (d) Overlay image of (b) and (c) [file 12864_2021_7674_MOESM11_ESM.pptx]

## Slide 1
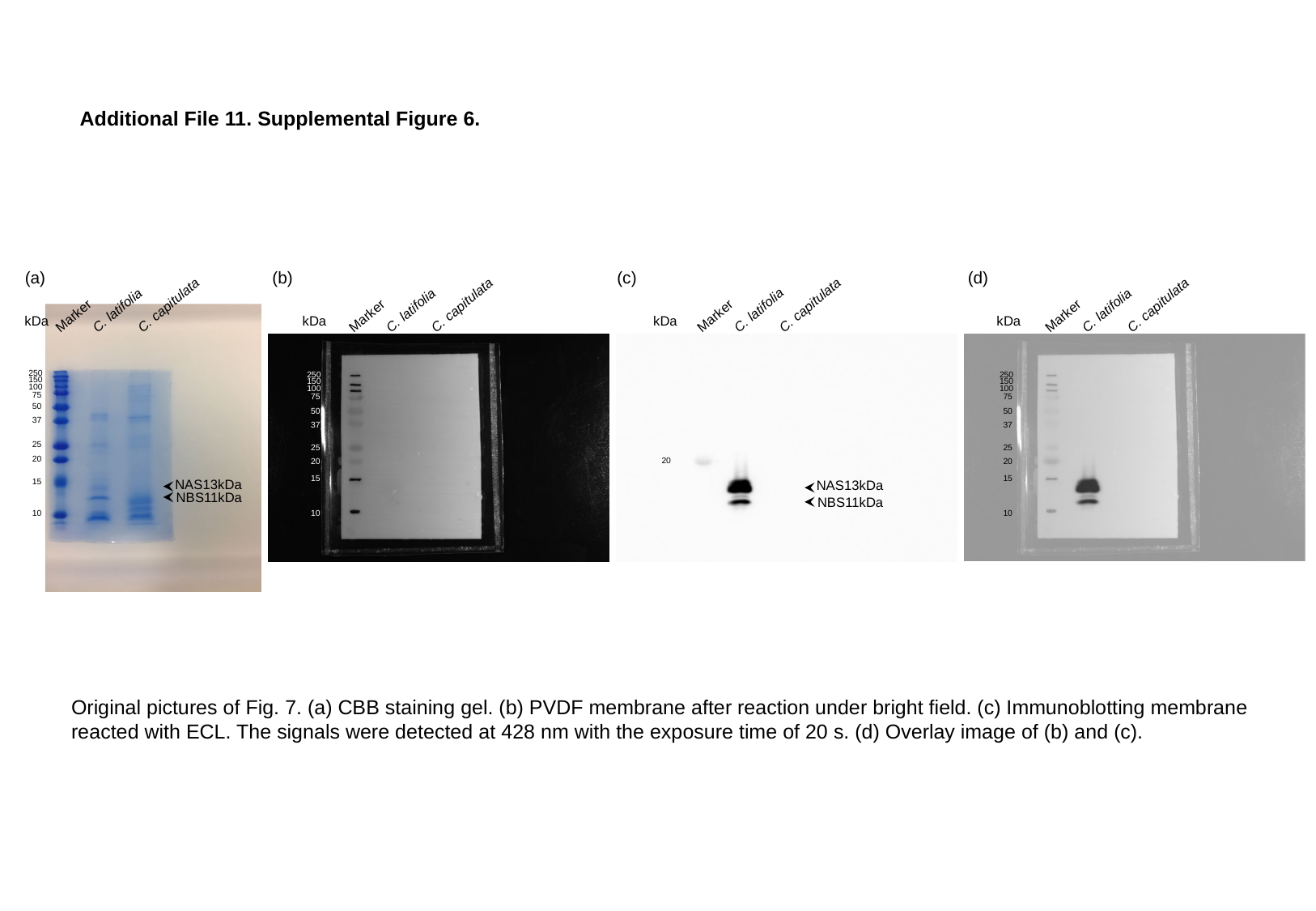

Additional File 11. Supplemental Figure 6.
(a)
(b)
(c)
(d)
C. capitulata
C. capitulata
C. capitulata
C. capitulata
C. latifolia
C. latifolia
C. latifolia
C. latifolia
kDa
kDa
kDa
kDa
Marker
Marker
Marker
Marker
250
250
250
150
150
150
100
100
100
75
75
75
50
50
50
37
37
37
25
25
25
20
20
20
20
15
15
NAS13kDa
15
NAS13kDa
NBS11kDa
NBS11kDa
10
10
10
Original pictures of Fig. 7. (a) CBB staining gel. (b) PVDF membrane after reaction under bright field. (c) Immunoblotting membrane reacted with ECL. The signals were detected at 428 nm with the exposure time of 20 s. (d) Overlay image of (b) and (c).
